# Porn use and men's and women's sexual performance: evidence from a large longitudinal sample

**DOI:** 10.1017/S003329172100516X

**Published:** 2023-05

**Authors:** Nicolas Sommet, Jacques Berent

**Affiliations:** 1 Centre LIVES, University of Lausanne, Lausanne, Switzerland; 2 School of Psychology (FPSE), University of Geneva, Geneva, Switzerland

**Keywords:** Fixed-effects regression, gender, pornography, sexual functioning, sexual satisfaction, sexual self-competence, sexuality

## Abstract

**Background:**

We examined whether young men and women differ in the relation between porn use and sexual performance (sexual self-competence, sexual functioning, and partner-reported sexual satisfaction).

**Methods:**

We conducted a three-wave longitudinal study (spanning 2015-16-17) that involved a very large number of men and women in their early 20s (100 000 + French-speaking individuals; 4000 + heterosexual couples).

**Results:**

The results revealed a twofold phenomenon. Among men, a higher frequency of porn use (wave 1) and increased porn use over time (waves 1–3) were associated with *lower* levels of sexual self-competence, impaired sexual functioning, and decreased partner-reported sexual satisfaction. In contrast, among women, higher and increasing frequencies of porn use were associated with *higher* levels of sexual self-competence, improved sexual functioning, and enhanced partner-reported sexual satisfaction (for some aspects).

**Conclusions:**

The findings reveal the irony that porn – a male-dominated industry that targets a male-dominated audience – is associated with the erosion of the quality of men's sex lives and the improvement of women's sex lives.

Young men and women differ in their porn use habits.^†^^1^ Men start using porn at an earlier age than women (Sinković, Štulhofer, & Božić, 2013), watch porn more often than women (Petersen & Hyde, 2010), and prefer hardcore over softcore videos (Hald, 2006). In this research, we further investigated porn-related gender differences by examining whether young men and women also differ in *the relation between porn use and sexual performance*.

Sexual performance can be studied based on different conceptualizations and operationalizations depending on the perspective taken. In this research, we distinguished among three sexual performance outcomes: (i) *sexual self-competence* (the sense of being sexually capable; Snell, 1998), (ii) *sexual functioning* (the degree of desire, arousal, erection/lubrication, orgasm, and satisfaction during sexual activities; Kalmbach, Ciesla, Janata, & Kingsberg, 2015), and (iii) *partner sexual satisfaction* (the quality of the sexual exchange/experience, and arguably the least biased measure of sexual performance; Štulhofer, Buško, & Brouillard, 2010b).

The research on the relations between the frequency of porn use and these three outcomes has shown mixed results (for narrative and systematic reviews, see Fisher & Kohut, 2017; Leonhardt, Spencer, Butler, & Theobald, 2019; Wright & Tokunaga, 2018). Such relations seem to be particularly equivocal in early adulthood, which is a critical time in the discovery of sexuality (Wallmyr & Welin, 2006). On the one hand, some authors have reported that porn use was associated with sexual performance concerns among young people, presumably because porn use sets unattainable standards of sexual comparison [e.g. not lasting as long as actors (for men) or not experiencing an orgasm as easily as actresses (for women); Goldsmith, Dunkley, Dang, & Gorzalka, 2017]. It is often thought that frequent porn use distorts beliefs about sexuality (Manning, 2006; Ward, 2016) and represents a threat to sexual self-competence, particularly for men (Morrison, Ellis, Morrison, Bearden, & Harriman, 2007). Accordingly, highly publicized authors have argued that porn was one of the root causes of ‘escalating, morphing sexual tastes, a range of sexual dysfunctions, and loss of attraction to real partners’ (Wilson, 2014; for other popular books, see Dines, 2010; Zimbardo & Coulombe, 2015).

On the other hand, some authors have warned against harm-focused research approaches that seek to demonstrate the adverse effects of porn use while disregarding its neutral or potentially beneficial effects (Campbell & Kohut, 2017). In fact, porn not only raises sexual performance concerns among young people but can also be used to acquire knowledge about certain sexual techniques [e.g. how to perform cunnilingus (for heterosexual men or lesbians) or fellatio (for heterosexual women or gay men); Peter & Valkenburg, 2016]. It was demonstrated that frequent porn use could actually broaden one's sexual horizons (Häggström-Nordin, Tydén, Hanson, & Larsson, 2009; Weinberg, Williams, Kleiner, & Irizarry, 2010) and foster sexual self-competence (Morrison, Harriman, Morrison, Bearden, & Ellis, 2004). Accordingly, some scholars have reported that the alleged negative effects of porn use on sexual quality or functioning lack robustness (Grubbs & Gola, 2019; Landripet & Štulhofer, 2015) and that these effects could sometimes be positive (Bőthe et al., 2021). It has even been suggested that porn use could serve as a therapeutic tool to treat hypoactive sexual disorder (Mollaioli, Sansone, Romanelli, & Jannini, 2018) or help couples suffering from sexual dissatisfaction (Watson & Smith, 2012).

Authors have suggested many possible moderators to account for the inconsistency in the relation between porn use and sexual performance-related outcomes (e.g. attitude toward pornography, context of porn use, relationship status; Leonhardt *et al*., 2019; Willoughby, Leonhardt, & Augustus, 2020). In this research, we drew on meta-analysis and/or literature reviews suggesting that one of the key moderators might be gender (Vaillancourt-Morel, Daspe, Charbonneau-Lefebvre, Bosisio, & Bergeron, 2019; Wright, Tokunaga, Kraus, & Klann, 2017). Because men and women hold different sexual preferences and gender roles (Petersen & Hyde, 2010; Stewart-Williams & Thomas, 2013), they tend to interpret, internalize, and apply different sexual scripts from porn (heuristics that tell them how to behave sexually; Wright, 2011), which could alter the relation between porn use and their sexual performance. To give a concrete example, because men have a higher sex drive than women, they may derive particular sexual guidelines from porn such as cutting foreplay, which may in turn lead to sexual callousness and erosion of relationship intimacy (for relevant research, see Bridges & Morokoff, 2011; Štulhofer, Buško, & Landripet, 2010a; see also Wright & Vangeel, 2019). In the same vein, men using porn may be more prone to developing sexual performance-related concerns from comparison to the actors and/or feel disappointed in their partner's inability (or lack of desire) to perform the sexual acts portrayed in porn (for relevant research, see Leonhardt & Willoughby, 2019; Sun, Bridges, Johnson, & Ezzell, 2016; Wright, Paul, Herbenick, & Tokunaga, 2021).

## Research questions and overview of the study

In this research, we aimed to test whether young men and women differ in terms of the relations between porn use and three sexual performance outcomes: What are the relations of porn use with men's and women's sexual self-competence (RQ_1_), sexual functioning (RQ_2_), and partner-reported sexual satisfaction (RQ_3_)?

Existing studies have tested similar moderation effects but have been limited in the sense that most of them had small sample sizes, relied on a cross-sectional design, and/or focused on one single subcomponent of sexual performance (e.g. Bridges & Morokoff, 2011; Morrison *et al*., 2007; Poulsen, Busby, & Galovan, 2013). To overcome this type of limitation, we recruited a sample of more than 100 000 participants, used a three-wave longitudinal design, and measured a comprehensive set of sexual performance outcomes. To build such a large sample, we collaborated with Mathieu Sommet, one of the most popular French YouTubers at the time of the research (with 1.6+ million subscribers). Mathieu posted an online video that invited his audience to complete our questionnaire, and voluntary participants were sent a similar follow-up questionnaire approximately one and then two years later. Although our sample was not nationally representative, Mathieu's audience had two key advantages as a target population: (i) youthfulness (his viewers were at a pivotal moment in their sexual development) and (ii) hyper-connectedness (his viewers had easy access to online porn). Note that Mathieu's then-channel ‘Salut Les Geeks’ (SLG) was a rather mainstream comedy channel (revolving around reviewing viral videos) that had nothing to do with sexual health. The deidentified data set, full materials (annotated questionnaires and codebooks), and Stata scripts to reproduce the findings are available on https://osf.io/nfbcp/.

## The SLG study

### Method

#### Ethics information

This study received approval from the Ethics Board of the University of Geneva.

#### Procedure and participants

Table 1 presents the sociodemographic and sexual characteristics for wave 1 (online Supplementary Table S1 presents similar information for waves 1–3).

**Table 1.** Description of the sociodemographic and sexual characteristics of the *wave 1 sample*

	*M*_♂_ (95% CI]	*M*_♀_ (95% CI]
Age	21.45 (21.41–21.48)	21.07 (21.04–21.11)
Percentage with a college education	34.81% (34.44–35.18)	36.66% (36.18–37.13)
Percentage from a French-speaking country (FR, CHE, BEL, LUX, CAN)	98.45% (98.35–98.54)	98.37% (98.25–98.49)
Sexual orientation (1 = *completely heterosexual*; 7 = *completely homosexual*) (measured using the so-called Kinsey scale; Kinsey, Pomeroy, & Martin, 1948)	1.66 (1.65–1.68)	2.15 (2.14–2.17)
Percentage who were nonvirgins	85.39% (85.11–85.66)	91.38% (91.10–91.65)
Age at first sexual intercourse^a^	16.76 (16.74–16.78)	16.56 (16.54–16.58)
Number of lifetime sexual partners^a^	6.52 (6.39–6.64)	6.16 (6.05–6.28)
Percentage in a relationship	47.68% (47.30–48.07)	63.82% (63.34–64.28)
Length of the relationship (in years, averaged between the two partners)^b^	2.61 (2.54–2.69)	2.62 (2.54–2.69)
Frequency of masturbation (past 6 months; 1 = *never*; 8 = *more than one time per day*)	5.26 (5.24–5.27)	3.36 (3.35–3.38)
Frequency of sexual intercourse (past 6 months; 1 = *never*; 8 = *more than one time per day*)	3.47 (3.45–3.48)	3.90 (3.88–3.91)
Knowledge about sexuality (percentage of correct answers on a true or false quiz) (measured using the 26-item Sexual Information subscale from Meston, Trapnell, & Gorzalka, 1998)	78.83% (78.76–78.89)	82.01% (81.94–82.09)
Social desirability (percentage of desirable answers on a true or false quiz) (measured using the 11-item short form of the Marlowe–Crowne scale (Form A; Reynolds, 1982)	51.30% (51.15–51.45)	49.03% (48.83–49.22)

a Only nonvirgins were considered; ^b^only participating partners were considered.

### Data collection

#### Wave 1

In June 2015, the French YouTuber Mathieu Sommet posted an online video that invited his followers to complete a questionnaire entitled ‘Sexual profile of adults’.^2^ A total of 171 462 participants (18+ year-olds) started the questionnaire, and 101 572 finished it. At the end of the questionnaire, the participants who were in relationships were asked to provide their own and their partner's birth dates (to identify and pair participating partners without using their names) and to forward the questionnaire to their partners.

#### Wave 2

Approximately one year later, the 47 575 participants who agreed to leave their email addresses at the end of the wave 1 questionnaire were sent a very similar follow-up questionnaire (*response rate*: 50.64%). Another year later, participants were again invited to complete the same follow-up questionnaire (*response rate*: 70.65%)

#### Samples/subsamples

For this research, we used the following (sub)samples:

- The *wave 1 sample* comprised 105 453 participants (61.45% men; 38.55% women) who provided complete data on our main predictor (the frequency of porn use) and first outcome of interest (sexual self-competence) for the first wave of data collection.
- The *waves 1–3 sample* comprised 21 898 participants (52.11% men; 47.89% women) who provided complete data on the same variables for at least two of the three waves of data collection. We excluded participants (2.80%) who reported inconsistent responses to the gender question (e.g. ‘woman’ in wave 1 and ‘man’ in wave 2).
- The *wave 1 couple subsample* comprised 8608 participating heterosexual partners whose sexual satisfaction information could be linked to one another. Nonheterosexual couples were excluded *a priori* because RQ_3_ applied to the effect of heteronormative porn on heterosexual romantic relationships.^3^ However, when these participants were included, the conclusions from the main analysis remained the same.
- The *waves 1–3 couple subsample* comprised 1002 participating heterosexual partners whose sexual satisfaction information could be linked to one another for at least two of the three waves of data collection. We used the same exclusion criteria used in the *wave 1 couple subsample*.

#### Variables

Table 2 presents the sample size, reliability, and descriptive statistics by gender for each variable. The items of each multi-item measure were averaged. Unless otherwise noted, all of the response scales ranged from 1 = *not at all* to 7 = *completely*.

**Table 2.** Description of the focal predictor, main outcomes, and alternative outcomes (robustness checks), along with sample size, reliability estimate (McDonalds' *Ω*), and descriptive statistics by gender

	Variable (sample size)	Number of item(s), sample item, response scale, and reference	*Ω*	*M*_♂_ (s.d.)	*M*_♀_ (s.d.)
Main outcomes	**Predictor.** Frequency of porn use (*N* = 105 453)	One item, i.e. ‘In the past six months, how often have you intentionally watched pornography on the Internet?’ (1 = *never*; 2 = *very rarely*; 3 = *rarely*; 4 = *occasionally*; 5 = *sometimes*; 6 = *regularly*; 7 = *often*; 8 = *very often*)	n/a	4.59 (1.80)	2.20 (1.37)
	**RQ_1_**. Sexual self-competence (*N* = 105 453)	Five items, e.g. ‘I would rate myself pretty favorably as a sexual partner’ (1 = *not at all*; 7 = *completely*) (Snell, 1998)	0.89	4.29 (1.50)	3.99 (1.51)
	**RQ_2_**. Sexual functioning (*N* = 105 402)	Six items: sexual desire, arousal, biological functioning, climax, discomfort, satisfaction (1 = *completely dysfunctional*; 7 = *completely functional*) (Isidori et al., 2010),	0.72	5.37 (1.16)	5.06 (1.13)
	**RQ_3_**. Partner-reported sexual satisfaction (*N* = 8608)	Five items – reported by participants' partners, e.g. ‘I am very satisfied with my sexual relationship’ (1 = *not at all*; 7 = *completely*) (Snell, 1998)	0.92	5.70 (1.46)	5.60 (1.50)
Alternative outcomes	**RQ_1_**. Sexual self-efficacy (*N* = 105 356)	Five items, e.g. ‘I have the ability to take care of any sexual needs and desires that I may have’ (1 = *not at all*; 7 = *completely*) (Snell, 1998)	0.89	4.74 (1.47)	4.64 (1.53)
	**RQ_1_**. Sexual anxiety (*N* = 105 384)	Five items, e.g. ‘I feel anxious when I think about the sexual aspects of my life’ (1 = *not at all*; 7 = *completely*) (Snell, 1998)	0.90	2.98 (1.76)	2.67 (1.68)
	**RQ_2_**. Sexual drive (*N* = 105 352)	Five items, e.g. ‘It doesn't take much to get me sexually excited’ (1 = *not at all*; 7 = *completely*) (Lippa, 2006)	0.79	4.91 (1.30)	4.42 (1.45)
	**RQ_3_**. Activity-specific sexual satisfaction (*N* = 8608)	Five items – partner-reported, e.g. ‘The variety of my sexual activities’ (1 = *not satisfied at all*; 8 = *completely satisfied*) (Štulhofer et al., 2010b)	0.82	6.38 (1.29)	6.22 (1.40)
	**RQ_3_**. Self-specific sexual satisfaction (*N* = 8608)	Six items – partner-reported, e.g. ‘The way I sexually react to my partner’ (1 = *not satisfied at all*; 8 = *completely satisfied*) (Štulhofer et al., 2010b)	0.82	6.26 (1.51)	6.65 (1.24)

*RQ_1–3_. Frequency of porn use.* We used the following item: ‘In the past six months, how often have you intentionally watched pornography on the Internet?’ (1 = *never*; 8 = *very often*). The item was not displayed for the participants who reported having never intentionally watched porn [7.83% of the sample; for these participants, the variable was recoded as ‘1’ (*never*)]. We followed the current recommendations (Kohut et al., 2020; Short, Black, Smith, Wetterneck, & Wells, 2012) and provided a definition of porn before presenting the item [‘any sexually explicit material (image/video) […] displaying a man/men's and/or a woman/women's genitalia with the aim of sexual arousal’].^4^

*RQ_1_. Sexual self-competence.* We used the sexual self-competence measure from the Multidimensional Sexual Self-Concept Questionnaire (MSSCQ; Snell, 1998; five items, e.g. ‘I am a pretty good sexual partner’).

*RQ_2_. Sexual functioning.* We adapted the items of the Sexual Function Index (Isidori et al., 2010), a six-item clinical tool that assesses sexual desire, sexual arousal, biological functioning (erection/lubrication), sexual climax, sexual satisfaction, and vaginal discomfort (for women) during sexual activities (for the exact wording of the items for men and women, see online Supplementary Table S3).

*RQ_3_. Partner-reported sexual satisfaction.* We used the sexual satisfaction measure from the MSSCQ (five items, e.g. ‘I am very satisfied with my sexual relationship’). For each wave, this variable was attached to the participating partner to create the partner-reported sexual satisfaction measure.

## Results

The full results can be found in Table 3.

**Table 3.** Standardized coefficients and 95% CIs of the cluster-adjusted regression models testing the effects of frequency of porn use as a function of gender (wave 1) and the fixed-effects panel regression models testing the effects of change in the frequency of porn use over time as a function of gender (waves 1–3) on sexual self-competence (RQ_1_), sexual functioning (RQ_2_), and partner-reported sexual satisfaction (RQ_3_)

		RQ_1_. Sexual self-competence		RQ_2_. Sexual functioning			RQ_3_. Partner-reported sexual satisfaction	
		*β*	95% CI	*β*	95% CI		*β*	95% CI
Gender (−0.5 = men; + 0.5 = women)	Wave 1 sample	−0.08***	(−0.09 to −0.07)	−0.13***	(−0.14 to −0.12)	Wave 1 couple subsample	−0.01	(−0.03 to 0.01)
Frequency of porn use		0.00	(−0.01 to 0.01)	−0.03***	(−0.04 to −0.03)		−0.03	(−0.05 to 0.00)
Gender × frequency of porn use		0.06***	(0.05–0.07)	0.05***	(0.05–0.06)		0.04***	(0.02–0.06)
		*β*	95% CI	*β*	95% CI		*β*	95% CI
Change in porn use over time	Wave 1–3 sample	0.00	(−0.02 to 0.01)	−0.02*	(−0.03 to −0.001)	Wave 1–3 couple subsample	−0.13**	(−0.22 to −0.05)
Gender × change in porn use over time		0.04***	(0.03–0.05)	0.07***	(0.06–0.08)		0.07*	(0.01–0.14)

*Notes*: All variables were standardized; ***p < 0.001; **p < 0.01; *p < 0.05.

### Overview of the two-step analytical strategy

#### Step #1. Cross-sectional analysis (between-participants estimates)

As a first step, we used the wave 1 data and built a regression model with standard errors (s.e.) adjusted for dyadic clustering (to address the issue of the interdependence of the residuals within couples). For each research question, we regressed our focal outcome *Y_i_* on gender, the frequency of porn use, and their interaction (Eq. 1):1
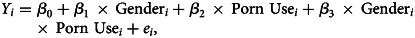
where *i* = 1, 2, …, *N* (participants) and *e_i_* is the error term.

#### Step #2. Longitudinal analysis (within-participants estimates)

The cross-sectional nature of the above analysis limited our ability to approach causality. In particular, the between-participants effect of porn use could be contaminated by unobserved heterogeneity, such as time-constant sexual preferences. Thus, as a second step, we used the waves 1–3 data and built a fixed-effects panel regression model that tested the effect of the *change* in the frequency of porn use over time as a function of gender. Fixed-effects panel regression is very popular in sociology or econometrics, but it has only recently received attention within psychology (McNeish & Kelley, 2019). This type of regression allows one to discard all observed *and* unobserved time-constant individual characteristics (eliminating all potential between-participant confounders) and obtain unbiased estimates of the pooled within-participant effects over time (Allison, 2009). Fixed-effects panel regression has been described as ‘one of the most powerful tools for studying causal processes using nonexperimental data’ (Osgood, 2010, p. 380) since causality is often inferred from the within-participant effects (however, for a discussion of its limitations, see Hill, Davis, Roos, & French, 2020). In our case, for each research question, we regressed the focal outcome *Y_it_* on the frequency of porn use and its interaction with gender (Eq. 2).^5^2
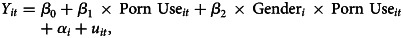
where *t* = 1, 2, 3 [waves], *α_i_* is participant fixed effects, and *u_it_* is the error term.

### RQ_1_: Porn use and men's and women's sexual self-competence

To test RQ_1_, we used sexual self-competence as the focal outcome.

#### Cross-sectional analysis

Our *wave 1 sample*-based cluster-adjusted regression model revealed that the relation between the frequency of porn use and sexual self-competence differed between men and women: For men, the higher the frequency of porn use, the *lower* the sexual self-competence, *β* = −0.06 (−0.07 to −0.05), *p* < 0.001 (numbers in round brackets represent 95% CIs); for women, the higher the frequency of porn use, the *higher* the sexual self-competence, *β* = 0.09 (0.07–0.11), *p* < 0.001. Following the current recommendations (Wright, 2021a), we repeated this and the subsequent cross-sectional analyses while controlling for the most commonly used sociodemographic and sexual characteristics (age, education, nationality, sexual orientation, number of lifetime sexual partners, relationship status, length of the relationship, frequency of masturbation, frequency of sexual intercourse, knowledge about sexuality, and social desirability), and the conclusions remained the same (online Supplementary Table S5).

#### Longitudinal analysis

**Fig. 1.**
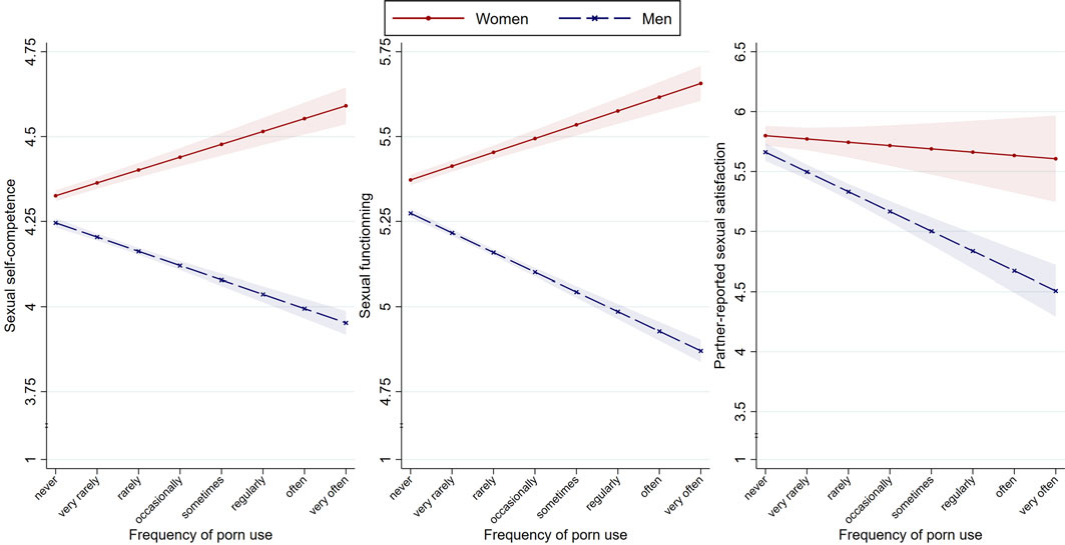
RQ_1–3_. Longitudinal effects of the frequency of porn use on sexual self-competence (RQ_1_, left panel), sexual functioning (RQ_2_, middle panel), and partner-reported sexual satisfaction in heterosexual couples (RQ_3_, right panel) among men and women. *Notes*: Shaded areas represent the s.e. of the means.

Our *waves 1–3 sample*-based fixed-effects panel regression model revealed that the effect of the change in the frequency of porn use on sexual self-competence differed between men and women: For men, an increase in the frequency of porn use over time was associated with a *reduction* in sexual self-competence, *β* = −0.08 (−0.11 to −0.06), *p* < 0.001; for women, an increase in the frequency of porn use over time was associated with an *increase* in sexual self-competence, *β* = 0.08 (0.05–0.11), *p* < 0.001 (Fig. 1, left panel). We repeated this and the subsequent longitudinal analyses while controlling for (time-varying) sociodemographic and sexual characteristics (relationship status, frequency of masturbation, frequency of sexual intercourse, and knowledge about sexuality) and period effects (wave dummies), and the conclusions remained the same (online Supplementary Table S6). Finally, given that the curvilinear effects of porn use on sexual outcomes had been documented (e.g. Wright, Steffen, & Sun, 2019), we repeated all main analyses while including the quadratic effect of frequency of porn use; the results – which are inconclusive – are presented in online Supplementary Table S7.

#### Robustness checks.

We performed two series of robustness checks.

##### Alternative outcomes

First, we repeated the main analyses using two alternative outcomes that were closely related to sexual self-competence, specifically, sexual self-efficacy and sexual anxiety [the full description of the measures is presented in the online Supplementary Materials, along with a PCA showing that the items loaded on different components (online Supplementary Table S2)]. The interactions were the same as in the main analysis. Both the frequency of porn use (wave 1) and the change in porn use over time (waves 1–3) had negative effects on men's sexual self-efficacy (whereas the effects were positive for women) and negative effects on men's sexual anxiety (whereas the effects were weaker or null for women; online Supplementary Table S8).

##### Alternative estimator

Second, we repeated the main longitudinal analysis using an alternative analytical approach: first-difference regression (Allison, 2009). Such an approach allows for the estimation of the change between two *consecutive* waves (rather than the overall within-participant change). The conclusions remained the same, increasing the plausibility (but not the certainty) that the findings are causal (online Supplementary Table S9). In this and the subsequent analyses, the conclusions of the two series of robustness checks remained similar when including our sets of control variables.

### RQ_2_: Porn use and men's and women's sexual self-functioning

To test RQ_2_, we used sexual functioning as the focal outcome.

#### Cross-sectional analysis

Our *wave 1 sample*-based cluster-adjusted regression model revealed that the relation between the frequency of porn use and sexual functioning differed between men and women: For men, the higher the frequency of porn use, the *lower* the sexual functioning, *β* = −0.09 (−0.09 to −0.08), *p* < 0.001; for women, the higher the frequency of porn use, the *higher* the sexual functioning, *β* = 0.05 (0.03–0.06), *p* < 0.001.

#### Longitudinal analysis

Our *waves 1–3 sample*-based fixed-effects panel regression model revealed that the effect of the change in the frequency of porn use on sexual functioning differed between men and women: For men, an increase in the frequency of porn use over time was associated with a *reduction* in sexual functioning, *β* = −0.10 (−0.13 to −0.08), *p* < 0.001; for women, the frequency of porn use over time was associated with an *increase* in sexual functioning, *β* = 0.07 (0.05–0.10), *p* < 0.001 (Fig. 1, middle panel).

#### Robustness checks

As in RQ_1_, we performed two series of robustness checks.

##### Alternative outcome

First, we repeated the main analyses using an alternative outcome that was closely related to sexual functioning, specifically, sexual drive (the full description of the measure is presented in online Supplementary Materials). The interaction was the same as in the main analysis. Both the frequency of porn use (wave 1) and the change in porn use over time (waves 1–3) had stronger positive effects on women's sexual drive than on men's sexual drive (online Supplementary Table S8).

##### Alternative estimator

Second, we repeated the main longitudinal analysis using our alternative estimator (first-difference regression). Again, the conclusions remained the same, increasing the plausibility (but not the certainty) that the findings are causal (online Supplementary Table S9).

### RQ_3_: Porn use and men's and women's partner-reported sexual satisfaction

To test RQ_3_, we used partner-reported sexual satisfaction as the focal outcome.

#### Cross-sectional analysis

Our *wave 1 couple subsample*-based cluster-adjusted regression model revealed that the relation between the frequency of porn use and partner-reported sexual satisfaction differed between men and women: For men, the higher the frequency of porn use, the *lower* their partner-reported sexual satisfaction, *β* = −0.08 (−0.10 to −0.04), *p* < 0.001; for women, the effect was not different from zero, *β* = 0.02 (−0.02 to 0.07), *p* = 0.272.

#### Longitudinal analysis

Our *waves 1–3 couple subsample*-based fixed-effects panel regression model revealed that the effect of the change in the frequency of porn use on partner-reported sexual satisfaction differed between men and women: For men, an increase in the frequency of porn use over time was associated with a *reduction* in their partner-reported sexual satisfaction, *β* = −0.23 (−0.32 to −0.13), *p* < 0.001; for women, the effect was not different from zero, *β* = −0.04 (−0.17 to 0.10), *p* = 0.587 (Fig. 1, right panel).

#### Robustness checks

As in RQs_1–2_, we performed two series of robustness checks.

##### Alternative outcomes

**Fig. 2.**
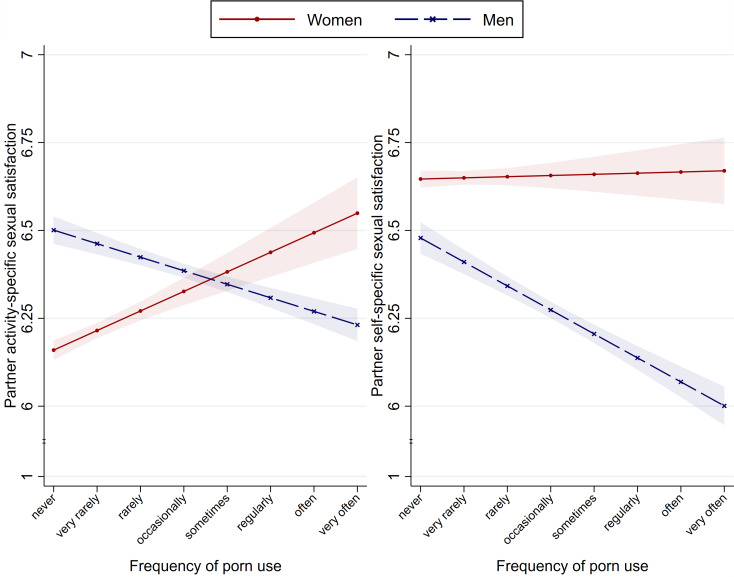
RQ_3_. Relation between the frequency of porn use and partner activity-specific (left panel) and self-specific (right panel) sexual satisfaction in heterosexual couples. *Notes*: Shaded areas represent the s.e. of the means.

First, we repeated the main analyses using two alternative outcomes that indicate two subdimensions of partner-reported sexual satisfaction, specifically, partner activity-specific sexual satisfaction (the quality of the sexual exchange) and partner self-specific (the quality of the personal sexual experience) sexual satisfaction [the full description of the measure is presented in the online Supplementary Materials, along with a PCA showing that the subdimension items loaded on different components (online Supplementary Table S4)]. In the *wave 1 couple subsample*, the frequency of porn use had a *negative* effect on men's partners' activity- and self-specific sexual satisfaction, whereas it had (i) a *positive* effect on women's partners' activity-specific sexual satisfaction and (ii) a *null* effect on women's partners' self-specific sexual satisfaction (Fig. 2). However, in the *waves 1–3 couple subsample*, we did *not* observe any significant interactions (online Supplementary Table S8).

##### Alternative estimator

Second, returning to our main outcome, we repeated the main longitudinal analyses using our alternative analytical approach (first-difference regression). Again, the conclusions remained the same, increasing the plausibility (but not the certainty) that the findings are causal (online Supplementary Table S9).

#### Supplementary exploratory analysis

We conducted exploratory analysis testing the idea that porn use could predict couple break ups; the results, which suggest that for women (but not for men), an increase in the frequency of porn use over time is associated with a decrease in the odds of a couple's break up, are presented in online Supplementary Materials.

## Discussion

The present research used a large-scale three-wave longitudinal sample to produce two robust sets of findings. For men, porn use is associated with lower sexual performance (lower sexual self-competence, sexual functioning, and partner-reported sexual satisfaction), whereas for women, porn use is associated with higher sexual performance (higher sexual self-competence, sexual functioning, and sexual partner-reported satisfaction – for some aspects).

### Interpretation of the findings

These two sets of findings could be interpreted in light of the extant literature. On the one hand, existing research reveals that porn can be a source of sexual inspiration that reinforces sexual permissiveness norms and widens the range of sexual practices and behaviors (Häggström-Nordin et al., 2009; Weinberg et al., 2010; Wright, Bae, & Funk, 2013). On the other hand, the existing research reveals that porn can also be a source of threatening upward sexual comparisons, particularly for men (Wright et al., 2021). For instance, the frequency of porn use predicts penis size dissatisfaction among men (whereas it does not predict genitalia/breast dissatisfaction among women; Cranney, 2015; but see Wright et al., 2017), and it predicts performance-related cognitive distraction during sexual activity among men (but not among women; Goldsmith et al., 2017). In the same vein, men watch more hardcore/paraphilic porn and less softcore/mainstream porn than women (Hald, 2006; Hald & Štulhofer, 2016), which may be associated with different sexual comparison processes and sexual outcomes (Leonhardt & Willoughby, 2019). These gender differences are consistent with our results. Among young men, the potentially inspiring nature of porn might be outweighed by its threatening nature: Porn use seemingly contributes to men's doubts about their sexual competence, the deterioration of their sexual functioning, and – in heterosexual couples – their partner-reported satisfaction. In contrast, among young women, the potentially inspiring nature of porn might outweigh its threatening nature: Porn use seemingly contributes to women's feelings of sexual competence, improvement in their sexual functioning, and – in heterosexual couples – some aspects of their partner-reported satisfaction.

### Implications

Our results are congruent with ideas sometimes expressed in the literature: (i) reducing porn use could help men to overcome sexual dysfunctions (Kirby, 2021) and (ii) increasing porn use could help women to improve their sexual lives (Mollaioli et al., 2018). However, proponents of these positions should bear in mind that, despite the robustness of our findings, the sex-specific effects of the frequency of porn use often had a low magnitude (0.05 ⩽ *β*s ⩽ 0.20), although they cannot be considered trivial (the strength of longitudinal associations is mechanically smaller than the strength of cross-sectional associations, and effects as small as *β* = 0.05 could still have important practical significance; Adachi & Willoughby, 2015). Accordingly, and contrary to what is often suggested in popular books on the psychology of pornography (e.g. Zimbardo & Coulombe, 2015), men who face sexual problems and choose to terminate porn use may experience only marginal improvements in their sexual lives (assuming that we can draw causal inferences from our findings); similarly, women who face sexual problems might be well advised not to consider porn use to be a sexual panacea.

### Limitations and conclusions

Three important limitations should be acknowledged.

First, >98% of our sample included residents from five French-speaking Western, educated, industrialized, rich, and democratic (WEIRD) countries (FR/BE/CH/LU/CA). Given that the effects of porn use are likely to vary from one cultural context to another (e.g. from nonreligious to religious contexts; Grubbs, Perry, Wilt, & Reid, 2019), replications with data from non-WEIRD countries are warranted.

Second, our method of data collection did not enable us to achieve national representativeness. Despite (i) our findings being robust to the inclusion of age, education, nationality, and sexual orientation controls; (ii) most demographics seeming not to be misrepresented (except gender in the wave 1 sample); and (iii) nonrepresentativeness being less of a concern when using within estimators (because individuals act as their own ‘controls’), replications with more representative data are also warranted.

Third, observational data cannot be used to draw causal inferences. However, we believe that causality should be assessed in terms of a ‘continuum of plausibility’ (Dunning, 2008) along which longitudinal evidence is located above cross-sectional evidence (but below experimental evidence; see also Grosz, Rohrer, & Thoemmes, 2020). In our case, given the consistencies between the results from the fixed-effects (focusing on within-participants change) and first-difference (focused on wave-to-wave change) regressions, we believe that causality is at least *plausible*. That being said, two alternative explanations – which we regard as less parsimonious in the case of a reversed interaction (for a related discussion, see Wright, 2021b) – cannot be formally excluded: (i) the presence of unobserved time-varying confounders (e.g. variations in well-being; see Kohut & Štulhofer, 2018) and (ii) reciprocal effects (e.g. for men, a decrease in sexual self-competence can cause an increase in porn use, and for women, the reverse could be true).

Despite these limitations, our findings reveal the irony that porn – a male-dominated industry that targets a male-dominated audience – is associated with the erosion of the quality of men's sex lives and the improvement of women's sex lives.

## Footnotes

† The notes appear after the main text.

## Notes

1 Porn is defined as any sexually explicit material (image/video) containing explicit exposure of the genitals with the aim of creating or enhancing sexual feelings or thoughts (Short et al., 2012).

2 Given the scale of the project, the survey included additional questions that are beyond the scope of this research. The materials and the data pertaining to these questions are available on https://osf.io/nfbcp/

3 We did not gather information regarding the participants' sexual identity (e.g. cis- or transgender).

4 The weekly time spent watching porn was also assessed. Because the interval differences between the adjacent categories of this variable were not constant, we used the frequency of porn use as our focal predictor. However, using the weekly time spent watching porn as an alternative predictor mostly led to the same conclusions (online Supplementary Table S10).

5 The main effect of gender (a time-constant variable) cannot be estimated because the fixed-effects panel regression focuses only on within-participants variation.

## Supplementary material

For supplementary material accompanying this paper visit https://doi.org/10.1017/S003329172100516X. click here to view supplementary material

## Open practices statement

The deidentified three-wave data set, full materials (i.e. annotated questionnaires and codebooks), and Stata scripts to reproduce the findings can be found at https://osf.io/nfbcp/.
