# Supplementary material for: Porn use and men's and women's sexual performance: evidence from a large longitudinal sample
Source: Psychol Med. 2022 Feb 9;53(7):3105–14. doi: 10.1017/S003329172100516X (PMC10235646; doi:10.1017/S003329172100516X)
Supplement: Supplementary file 1 [file S003329172100516Xsup001.docx]

Supplemental Materials for
Porn Use and Men’s and Women’s Sexual Performance:
Evidence from a Large Longitudinal Sample

This document is organized as follows:

● ***Method (p. 2)***

• P. 2. **Table S1**. Description of the sociodemographic and sexual characteristics of the *Waves 1-3 Samples*

● ***RQ_1_: Sexual Self-Competence (pp. 3-4)***

• P. 3. RQ_1_, description of the alternative sexual self-evaluation measures used in the 1^st^ robustness check: sexual self-efficacy and sexual anxiety

• P. 4. **Table S2**. RQ_1_ – Principal component analysis: sexual self-competence, self-efficacy, and anxiety items.

● ***RQ_2_: Sexual Functioning (pp. 5-6)***

• P. 5. RQ_2_, description of the alternative sexual functioning measure used in the 1^st^ robustness check: sexual drive

• P. 6. **Table S3**. RQ_2_ – Details of the items of our main sexual functioning measure (wording, response scale, and descriptive statistics by gender).

● ***RQ_3_: Partner-Reported Sexual Satisfaction (pp. 7-9)***

• P. 7. RQ_3_, description of the alternative partner-reported sexual satisfaction used in the 1^st^ robustness check: partner activity-specific and self-specific sexual satisfaction.

• P. 8. **Table S4**. RQ_3_ – Principal component analysis: partner activity-specific and self-specific sexual satisfaction items.

• P. 9. RQ_3_, supplementary exploratory analysis testing the idea that porn use could predict couple breaks ups.

● ***RQ_1-3_: Sexual Self-Competence, Functioning, and Partner Sexual Satisfaction (pp. 10-16)***

• P. 11. **Table S5**. RQ_1-3_ – Interindividual control variables: Repeating the main cross-sectional analyses while controlling for interindividual differences.

• P. 12. **Table S6**. RQ_1-3_ – Time-varying control variables: Repeating the main longitudinal analyses while controlling for time-varying covariates.

• P. 13. **Table S7**. RQ_1-3_ – Curvilinear effects: Repeating the main cross-sectional and longitudinal analyses while including the quadratic effect of porn use.

• P. 14. **Table S8**. RQ_1-3_ – 1^st^ robustness check (alternative outcomes): Repeating the main analyses while using alternative outcomes.

• P. 15. **Table S9**. RQ_1-3_ – 2^nd^ robustness check (alternative estimator): Repeating the main analyses while using first-difference regression.

• P. 16. **Table S10**. RQ_1-3_ – Alternative predictor: Repeating the main analyses while using weekly time spent watching porn as an alternative predictor.

### **Table S1**

Description of the sociodemographic and sexual characteristics of the *Waves 1-3 Samples*

|  | Wave 1 | Wave 2 | Wave 3 |
| --- | --- | --- | --- |
| Percentage of women | 38.55% [38.25, 38.84] | 47.94% [47.26, 48.62] | 51.36% [50.57, 52.15] |
| Age^§^ | 21.30 [21.28, 21.33] | 22.54 [22.49, 22.60] | 23.61 [23.55, 23.68] |
| Percentage with a college education^§^ | 35.52% [35.23, 35.81] | 38.66% [38, 39.33] | 40.31% [39.54, 41.09] |
| Percentage from a French-speaking country (FR, CHE, BEL, LUX, CAN)^§^ | 98.42% [98.35, 98.50] | 98.64% [98.48, 98.80] | 98.69% [98.52, 98.87] |
| Sexual orientation (1 = *Completely heterosexual*; 7 = *Completely homosexual*) | 1.85 [1.84, 1.86] | 2.09 [2.07, 2.11] | 2.15 [2.12, 2.17] |
| Percentage who were nonvirgins | 87.70% [87.50, 87.90] | 91.43% [91.05, 91.81] | 93.35% [92.96, 93.75] |
| Age at first sexual intercourse^*§^ | 16.68 [16.67, 16.70] | 17.03 [16.99, 17.06] | 17.14 [17.10, 17.18] |
| Number of lifetime sexual partners^*^ | 6.37 [6.29, 6.46] | 7.86 [7.63, 8.09] | 9.07 [8.78, 9.36] |
| Percentage in a relationship | 53.9% [53.60, 54.20] | 58.67% [58.00, 59.34] | 62.63% [61.86, 63.4] |
| Length of the relationship (in years, averaged between the two partners)^†^ | 2.62 [2.54, 2.69] | 3.99 [3.82, 4.16] | 5.22 [4.96, 5.48] |
| Frequency of masturbation (past six months; 1 = *never*; 8 = *>1 per day*) | 4.53 [4.52, 4.54] | 4.56 [4.54, 4.59] | 4.45 [4.42, 4.48] |
| Frequency of sexual intercourse (past six months; 1 = *never*; 8 = *>1 per day*) | 3.64 [3.63, 3.65] | 3.59 [3.57, 3.62] | 3.50 [3.48, 3.53] |
| Knowledge about sexuality (% of correct answers on a true or false quiz) | 80.06% [80.01, 80.11] | 82.25% [82.16, 82.34] | 82.89% [82.79, 82.99] |
| Social desirability (percentage of desirable answers on a true or false quiz)^§^ | 50.42% [50.30, 50.54] | 50.77% [50.50, 51.03] | 50.96% [50.65, 51.27] |

*Notes*: ^*^only nonvirgins were considered; ^†^only participating partners were considered; ^§^these variables were only measured at Wave 1.

## **RQ_1_: Sexual Self-Competence**

### **Alternative Sexual Self-Evaluation Outcomes:** **Sexual Self-Efficacy and Anxiety**

We used the sexual self-efficacy measure (five items, e.g., “I have the ability to take care of any sexual needs and desires that I may have”) and the sexual anxiety measure (five items, e.g., “I have the ability to take care of any sexual needs and desires that I may have”) from the MSSCQ (Snell, 1998). All the items that measured RQ_1_’s main and alternative outcomes coherently loaded on three different components (for the results of the principal component analysis, see Table S1).

### **Table S2**

RQ_1_: Principal component analysis (Oblimin rotation) of the items measuring sexual self-competence (component C1), sexual anxiety (C2), and sexual self-efficacy (C3).

|  | C1 | C2 | C3 |
| --- | --- | --- | --- |
| Je m’évaluerais assez favorablement en tant que partenaire sexuel(-le) [I would rate myself pretty favorably as a sexual partner] | **.89** | -.37 | .49 |
| J’ai le sentiment d’être un(e) assez bon(-ne) partenaire sexuel(-le) [I am a pretty good sexual partner] | **.89** | -.43 | .51 |
| J’ai confiance en moi en tant que partenaire sexuel(le) [I am confident about myself as a sexual partner] | **.86** | -.46 | .52 |
| Je pense être meilleur(e) dans les rapports sexuels que la plupart des gens [I am better at sex than most other people] | **.79** | -.19 | .36 |
| Si j’étais amené(e) à avoir un rapport sexuel imprévu, je serais très confiant(e) [I would be very confident in a sexual encounter] | **.70** | -.34 | .38 |
| Je ressens de l’anxiété quand je pense à ma vie sexuelle [I feel anxious when I think about the sexual aspects of my life] | -.36 | **.89** | -.35 |
| Je suis nerveux(-euse) lorsque je pense à ma vie sexuelle [I feel nervous when I think about the sexual aspects of my life] | -.37 | **.87** | -.35 |
| Penser à ma vie sexuelle provoque en moi un sentiment de malaise [Thinking about the sexual aspects of my life leaves me with an uneasy feeling] | -.38 | **.83** | -.38 |
| Je me fais du souci par rapport à ma vie sexuelle [I am worried about the sexual aspects of my life] | -.32 | **.83** | -.35 |
| J’ai l’habitude de m’inquiéter concernant ma vie sexuelle [I usually worry about the sexual aspects of my life] | -.28 | **.82** | -.29 |
| J’ai la capacité de répondre à mes besoins et désirs sexuels [I have the capability to take care of my own sexual needs and desires] | .45 | -.37 | **.89** |
| J’ai la capacité de pourvoir aux besoins et désirs sexuels que je peux avoir [I have the ability to take care of any sexual needs and desires that I may have] | .49 | -.36 | **.85** |
| Je suis suffisamment compétent(e) pour garantir la satisfaction de mes besoins sexuels [I am competent enough to make sure that my sexual needs are fulfilled] | .58 | -.37 | **.84** |
| Je suis capable de faire face à mes propres besoins et désirs sexuels [I am able to cope with and to handle my own sexual needs and wants] | .34 | -.29 | **.82** |
| J’ai les aptitudes et capacités pour m’assurer une vie sexuelle satisfaisante [I have the skills and ability to ensure rewarding sexual behaviors for myself] | **.68** | -.45 | **.73** |
| Percent of variance explained | 47.3 | 14.8 | 9.4 |

*Notes*: The items loading above .6 are shown in bold.

## **RQ_2_: Sexual Self-Functioning**

### **Alternative Sexual Functioning Outcomes:** **Sexual Drive**

We used the sexual drive measure (five items, e.g., “It doesn’t take much to get me sexually excited”) from the Sexual Attitudes and Feeling Scale (Lippa, 2006).

### **Table S3**

RQ_2_: Details of the items of our sexual functioning measure (wording, response scale, and descriptive statistics by gender).

| Variable (sample size) | Items and scale | *M*_♂_ (SD) | *M*_♀_ (SD) |
| --- | --- | --- | --- |
| Sexual desire (*N* = 95,203) | “How would you rate your level (degree) of sexual desire or interest?” (1 = *none at all*; 7 = *very high*) | 5.45 (1.29) | 5.14 (1.43) |
| Sexual arousal (*N* = 95,511) | “How would you rate your level of sexual arousal (‘turn on’) during sexual activity or intercourse?” (1 = *none at all*; 7 = *very high*) | 5.63 (1.34) | 5.48 (1.40) |
| Biological functioning (*N* = 95,515) | “How often did your penis become erect [/did you become lubricated (‘wet’)] when trying to engage in sexual activity or intercourse?” (1 = *never*; 7 = *always*) | 5.79 (1.37) | 6.55 (0.95) |
| Sexual climax (*N* = 96,431) | “When you had sexual stimulation or intercourse, how often did you reach orgasm (climax)?” (1 = *never*; 7 = *always*) | 5.82 (1.47) | 4.45 (1.93) |
| Sexual satisfaction  (*N* = 105,139) | “I am very satisfied with my sexual life” (1 = *not at all*; 7 = *completely*) | 4.15 (2.25) | 4.76 (2.09) |
| Sexual discomfort  (*N* = 35,708) | “How often did you experience discomfort or pain during vaginal penetration?” (1 = *never*; 7 = *always*; for women only) | n/a | 5.24 (1.51) |

## **RQ_3_: Partner-Reported Sexual Satisfaction**

### **Alternative Partner-Reported Sexual Satisfaction Outcomes:** **Partner Activity-Specific and Self-Specific Sexual Satisfaction.**

We used the activity-specific sexual satisfaction measure (six items, “The variety of my sexual activities”) and the self-specific sexual satisfaction measure (six items, “The way I sexually react to my partner”) from the New Sexual Satisfaction Scale (NSSS; Štulhofer et al., 2010). The response scale ranged from 1 = *not satisfied at all* to 8 = *completely satisfied*. All but one item of the NSSS coherently loaded on two components (the problematic item was disregarded; for the results of the principal component analysis, see Table S3). For each wave, this variable was also attached to the participating partner to create the partner-reported activity-specific and self-specific sexual satisfaction measures.

### **Table S4**

RQ_3_: Principal component analysis (Oblimin rotation) of the items measuring partner activity-specific (component C1) and self-specific (C2) sexual satisfaction^6^.

|  | **C1** | **C2** |
| --- | --- | --- |
| La capacité de mon(ma) partenaire d’avoir un orgasme  [My partner’s ability to orgasm] (activity3) | **0.73** | -0.20 |
| L’ouverture émotionnelle de mon(ma) partenaire durant le sexe  [My partner’s emotional opening up during sex] (activity2) | **0.73** | -0.41 |
| La diversité de mes activités sexuelles  [The variety of my sexual activities] (activity5) | **0.72** | -0.55 |
| La créativité sexuelle de mon(ma) partenaire  [My partner’s sexual creativity] (activity4) | **0.71** | -0.46 |
| Le plaisir que je procure à mon(ma) partenaire  [The pleasure I provide to my partner] (self6) | **0.71** | -0.35 |
| L’équilibre entre ce que je donne et ce que je reçois dans le sexe  [The balance between what I give and receive in sex] (activity1) | **0.67** | -0.59 |
| La fréquence de mes activités sexuelles  [The frequency of my sexual activity] (activity6) | **0.61** | -0.48 |
| Mon "laisser-aller" et ma capacité à m’abandonner au plaisir pendant le sexe  [My “letting go” and surrender to sexual pleasure during sex] (self2) | 0.41 | **-0.79** |
| La façon dont je réagis sexuellement à mon(ma) partenaire  [The way I sexually react to my partner] (self3) | 0.51 | **-0.79** |
| La qualité de mes orgasmes  [The intensity of my sexual arousal] (self1) | 0.37 | **-0.78** |
| Le fonctionnement sexuel de mon corps  [My body’s sexual functioning] (self4) | 0.33 | **-0.76** |
| Mon humeur après une activité sexuelle  [My mood after sexual activity] (self5) | 0.50 | **-0.67** |
| Percent of variance explained | 44.22 | 10.43 |

*Notes*: Partner activity-specific sexual satisfaction was computed using the mean of activity1-activity6; partner self-specific sexual satisfaction was computed using the mean of self1-self5; the item self6 was disregarded because it did not load on the relevant component; items loading above .6 are shown in bold.

### **Supplementary Exploratory Analysis.**

Compared to the RQs_1-2_ findings, the RQ_3_ robustness checks were somewhat less consistent. The *longitudinal* interaction effect was not replicated when we used our alternative outcomes. Compared to the other samples, an obvious difference in the Waves 1-3 Couple Subsample was the steep decrease in the sample size (and related statistical power). One of the most important factors for this decrease in sample size was couple break-ups (33.56% of the couples broke up during the study and could no longer be considered in the longitudinal analysis). Out of curiosity, we explored the possibility that porn use could have played a role in these break-ups (if later reported by at least one partner). A first-difference regression that used heterosexual couples as the units of analysis (*N* = 791) revealed that, for men, a change in the frequency of porn use did not significantly predict the odds of a couple’s break-up, *OR* = 0.97 [0.86, 1.08], *p* = .560, whereas for women, an increase in the frequency of porn use by one SD predicted a 12% *decrease* in the odds of a couple’s break-up, *OR* = 0.89 [0.82, 0.98], *p* = .014. These findings—which are exploratory and should therefore be interpreted with caution—echo the findings from Perry and David (2017), who documented that the effect of porn use on the odds of breakup differed between men and women (over a six-year period), although the authors did not observe *beneficial* effects of porn use among women (for additional relevant research on porn use and marital separation or divorce, see Perry, 2018; Perry & Schleifer, 2018).

## **RQ_1-3_: Sexual Self-Competence, Functioning, and Partner-Reported Sexual Satisfaction**

### **Table S5**

RQ_1-3_ – Interindividual control variables: Standardized coefficients and 95% CIs of the cluster-adjusted regression models testing the effects of the frequency of porn use as a function of gender (Wave 1) on sexual self-competence (RQ_1_), sexual functioning (RQ_2_), and partner-reported sexual satisfaction (RQ_3_) while controlling for interindividual differences.

|  | | **RQ_1_.** Sexual  self-competence | | **RQ_2_.** Sexual functioning | |  | **RQ_3_.** Partner-reported sexual satisfaction | |
| --- | --- | --- | --- | --- | --- | --- | --- | --- |
|  |  | β | 95% CI | β | 95% CI |  | β | 95% CI |
| Gender (-0.5 = men; +0.5 = women) | Wave 1 Sample | -0.10^***^ | [-0.11, -0.09] | -0.18^***^ | [-0.19, -0.17] | Wave 1 Couple Subsample | -0.02 | [-0.04, 0.01] |
| Frequency of porn use |  | 0.02^***^ | [0.01, 0.03] | 0.03^***^ | [0.02, 0.04] |  | -0.01 | [-0.05, 0.03] |
| Gender × porn use |  | 0.03^***^ | [0.02, 0.03] | 0.02^***^ | [0.02, 0.03] |  | 0.02^*^ | [0.002, 0.05] |
| Age |  | 0.01^***^ | [0.01, 0.02] | -0.03^***^ | [-0.04, -0.03] |  | -0.05^**^ | [-0.07, -0.02] |
| Education (1 = College-educated) |  | 0.01^***^ | [0.01, 0.02] | 0.00 | [-0.00, 0.01] |  | 0.01 | [-0.01, 0.03] |
| Nationality (1 = French-speaking country) |  | -0.02^***^ | [-0.02, -0.01] | 0.00 | [-0.01, 0.00] |  | -0.02 | [-0.04, 0.00] |
| Sexual orientation |  | -0.02^***^ | [-0.03, -0.02] | 0.00 | [-0.00, 0.01] |  | -0.02 | [-0.04, 0.00] |
| Number of lifetime sexual partners |  | 0.13^***^ | [0.12, 0.15] | 0.02^***^ | [0.02, 0.03] |  | 0 | [-0.02, 0.02] |
| Relationship status (1 = *in a relationship*) |  | 0.05^***^ | [0.04, 0.06] | 0.18^***^ | [0.18, 0.19] |  | n/a | n/a |
| Length of the relationship |  | n/a | n/a | n/a | n/a |  | -0.04^**^ | [-0.07, -0.01] |
| Frequency of masturbation (past 6 months) |  | 0.07^***^ | [0.06, 0.08] | 0.07^***^ | [0.06, 0.08] |  | -0.01 | [-0.04, 0.02] |
| Frequency of sexual intercourse (past 6 months) |  | 0.44^***^ | [0.43, 0.44] | 0.47^***^ | [0.47, 0.48] |  | 0.36^***^ | [0.34, 0.39] |
| Knowledge about sexuality |  | -0.01^***^ | [-0.02, -0.01] | 0.04^***^ | [0.03, 0.04] |  | 0.02 | [-0.00, 0.04] |
| Social desirability |  | 0.04^***^ | [0.03, 0.04] | 0.04^***^ | [0.04, 0.05] |  | 0.02 | [-0.01, 0.04] |

*Notes*: For the description of the control variable, see Table 1; n/a means “not applicable” (length of the relationship is a couple-specific variable and relationship status does not vary in the couple subsample); ^***^*p* < .001, ^**^*p* < .01, ^**^*p* < .05.

### **Table S6**

RQ_1_-_3_ – Time-varying control variables: Standardized coefficients and 95% CIs of the fixed-effects panel regression models testing the effects of the change in the frequency of porn use over time as a function of gender (Waves 1-3) on sexual self-competence (RQ_1_), sexual functioning (RQ_2_), and partner-reported sexual satisfaction (RQ_3_) while controlling for relevant time-varying covariates and period effects.

|  | | **RQ_1_.** Sexual  self-competence | | **RQ_2_.** Sexual functioning | |  | **RQ_3_.** Partner-reported sexual satisfaction | |
| --- | --- | --- | --- | --- | --- | --- | --- | --- |
|  |  | β | 95% CI | β | 95% CI |  | β | 95% CI |
| Change in porn use over time | Wave 1 Sample | -0.01 | [-0.02, 0.01] | 0.02 | [-0.00, 0.03] | Waves 1-3 Couple Subsample | -0.07 | [-0.16, 0.02] |
| Gender × change in porn use over time |  | -0.02^***^ | [-0.03, -0.01] | -0.03^***^ | [-0.05, -0.02] |  | -0.07^*^ | [-0.13, -0.01] |
| Relationship status (1 = *in a relationship*) |  | 0.05^***^ | [0.04, 0.06] | 0.13^***^ | [0.12, 0.14] |  | n/a | n/a |
| Frequency of masturbation (past 6 months) |  | 0.04^***^ | [0.03, 0.06] | 0.09^***^ | [0.07, 0.11] |  | 0.05 | [-0.03, 0.13] |
| Frequency of sexual intercourse (past 6 months) |  | 0.21^***^ | [0.20, 0.22] | 0.43^***^ | [0.42, 0.44] |  | 0.26^***^ | [0.21, 0.32] |
| Knowledge about sexuality |  | 0 | [-0.01, 0.00] | 0.01^**^ | [0.00, 0.02] |  | -0.02 | [-0.07, 0.02] |
| Period effects |  |  |  |  |  |  | 0 | [0.00, 0.00] |
| Wave 1 vs. Wave 2 |  | 0.05^***^ | [0.04, 0.06] | -0.02^**^ | [-0.03, -0.01] |  | -0.23^***^ | [-0.29, -0.17] |
| Wave 1 vs. Wave 3 |  | 0.11^***^ | [0.10, 0.12] | -0.02^**^ | [-0.04, -0.01] |  | -0.34^***^ | [-0.43, -0.25] |

*Notes*: All variables were standardized; ^***^*p* < .001, ^**^*p* < .01; ^*^*p* < .05.

### **Table S7**

Standardized coefficients and 95% CIs of the cluster-adjusted regression models testing the linear and quadratic effects of frequency of porn use as a function of gender (Wave 1) and the fixed-effects panel regression models testing the linear and quadratic effects of change in the frequency of porn use over time as a function of gender (Waves 1-3) on sexual self-competence (RQ_1_), sexual functioning (RQ_2_), and partner-reported sexual satisfaction (RQ_3_).

|  | | **RQ_1_.** Sexual  self-competence | | **RQ_2_.** Sexual functioning | |  | **RQ_3_.** Partner-reported sexual satisfaction | |
| --- | --- | --- | --- | --- | --- | --- | --- | --- |
|  |  | β | 95% CI | β | 95% CI |  | β | 95% CI |
| Gender (-0.5 = men; +0.5 = women) | Wave 1  Sample | 0.15^***^ | [0.13, 0.17] | 0.27^***^ | [0.25, 0.29] | Wave 1 Couple Subsample | 0.02 | [-0.04, 0.07] |
| Frequency of porn use |  | 0.23^***^ | [0.19, 0.27] | 0.21^***^ | [0.18, 0.25] |  | 0.13^**^ | [0.03, 0.23] |
| Gender × frequency of porn use |  | -0.15^***^ | [-0.17, -0.13] | -0.16^***^ | [-0.17, -0.14] |  | -0.10^***^ | [-0.17, -0.04] |
| Frequency of porn use^2^ |  | -0.04^*^ | [-0.08, -0.01] | 0.02 | [-0.01, 0.05] |  | -0.01 | [-0.10, 0.07] |
| Gender × frequency of porn use^2^ |  | 0.03^**^ | [0.01, 0.05] | 0.00 | [-0.02, 0.01] |  | 0.01 | [-0.04, 0.06] |
|  | | β | 95% CI | β | 95% CI |  | β | 95% CI |
| Change in porn use over time | Wave 1-3 Sample | 0.14^***^ | [0.09, 0.18] | 0.26^***^ | [0.20, 0.32] | Wave 1-3 Couple Subsample | 0.2 | [-0.10, 0.51] |
| Gender × change in porn use over time |  | -0.10^***^ | [-0.12, -0.07] | -0.19^***^ | [-0.23, -0.15] |  | -0.22^*^ | [-0.40, -0.03] |
| Change in porn use over time^2^ |  | -0.04^*^ | [-0.08, -0.005] | -0.03 | [-0.08, 0.02] |  | 0.17 | [-0.07, 0.42] |
| Gender × change in porn use over time^2^ |  | 0.02 | [-0.00, 0.04] | 0.02 | [-0.00, 0.05] |  | -0.08 | [-0.22, 0.05] |

*Notes*: All variables were standardized; ^***^*p* < .001, ^**^*p* < .01; ^*^*p* < .05.

### **Table S8**

RQ_1-3_ – 1^st^ robustness check (alternative outcomes): Standardized coefficients and 95% CIs of the cluster-adjusted regression models testing the effects of the frequency of porn use as a function of gender (Wave 1) and the fixed-effects panel regression models testing the effects of the change in the frequency of porn use over time as a function of gender (Waves 1-3) on the following alternative outcomes: sexual self-efficacy and sexual anxiety (RQ_1_), sexual drive (RQ_2_), and partner activity-specific and self-specific sexual satisfaction (RQ_3_).

|  | | **RQ_1_.** Sexual  self-efficacy | | **RQ_1_.** Sexual anxiety | | **RQ_2_.** Sexual  drive | |  | **RQ_3_.** Partner sexual satisfaction | | | |
| --- | --- | --- | --- | --- | --- | --- | --- | --- | --- | --- | --- | --- |
|  |  |  |  |  |  |  |  |  | activity-specific | | self-specific | |
|  |  | β | 95% CI | β | 95% CI | β | 95% CI |  | β | 95% CI | β | 95% CI |
| Gender (-0.5 = men; +0.5 = women) | Wave 1 Sample | 0.02^***^ | [0.01, 0.03] | -0.02^***^ | [-0.02, -0.01] | 0.02^***^ | [0.01,0.03] | Wave 1 Couple Subsample | -0.05^***^ | [-0.08, -0.03] | 0.11^***^ | [0.09, 0.14] |
| Frequency of porn use |  | 0.03^***^ | [0.02, 0.03] | 0.15^***^ | [0.14, 0.16] | 0.31^***^ | [0.30,0.32] |  | 0.01 | [-0.02, 0.04] | -0.04^**^ | [-0.07, -0.02] |
| Gender × frequency of porn use |  | 0.12^***^ | [0.11, 0.12] | -0.04^***^ | [-0.05, -0.04] | 0.04^***^ | [0.03,0.05] |  | 0.05^***^ | [0.03, 0.08] | 0.04^***^ | [0.02, 0.06] |
| Simple slope for men |  | -0.09^***^ | [-0.09, -0.08] | 0.19^***^ | [0.18, 0.20] | 0.27^***^ | [0.26, 0.28] |  | -0.05^***^ | [-0.08, -0.02] | -0.09^***^ | [-0.13, -0.06] |
| Simple slope for women |  | 0.21^***^ | [0.19, 0.22] | 0.09^***^ | [0.07, 0.10] | 0.37^***^ | [0.36, 0.38] |  | 0.08^***^ | [0.03, 0.12] | 0 | [-0.04, 0.05] |
|  | | β | 95% CI | β | 95% CI | β | 95% CI |  | β | 95% CI | β | 95% CI |
| Change in porn use over time | Wave 1-3 Sample | 0.00 | [-0.02, 0.01] | 0.06^***^ | [0.05, 0.08] | 0.16^***^ | [0.15, 0.18] | Wave 1-3 Subsample | -0.08^*^ | [-0.17, -0.001] | -0.06 | [-0.13, 0.02] |
| Gender × change in porn use over time |  | 0.07^***^ | [0.06, 0.09] | -0.05^***^ | [-0.06, -0.04] | 0.03^***^ | [0.02, 0.04] |  | 0.04 | [-0.02, 0.11] | 0.05 | [-0.01, 0.10] |
| Simple slope for men |  | -0.10^***^ | [-0.12, -0.08] | 0.12^***^ | [0.10, 0.14] | 0.12^***^ | [0.11, 0.14] |  | -0.14^**^ | [-0.23, 0.04] | -0.11^**^ | [-0.20, -0.03] |
| Simple slope for women |  | 0.10^***^ | [0.07, 0.12] | -0.01 | [-0.03, 0.02] | 0.21^***^ | [0.19, 0.23] |  | -0.3 | [-0.16, 0.10] | 0 | [-0.12, 0.12] |

*Notes*: All variables were standardized; ^***^*p* < .001, ^**^*p* < .01, ^**^*p* < .05.

### **Table S9**

RQ_1_-RQ_3_ – 2^nd^ robustness check (alternative estimator): Standardized coefficients and 95% CIs of the first-difference regression models testing the effects of the change in the frequency of porn use over time as a function of gender (Waves 1-3) on sexual self-competence (RQ_1_), sexual functioning (RQ_2_), and partner-reported sexual satisfaction (RQ_3_).

|  | | **RQ_1_.** Sexual  self-competence | | **RQ_2_.** Sexual functioning | |  | **RQ_3_.** Partner-reported sexual satisfaction | |
| --- | --- | --- | --- | --- | --- | --- | --- | --- |
|  |  | β | 95% CI | β | 95% CI |  | β | 95% CI |
| Change in porn use over time  (first-difference operator) | Wave 1-3 Sample | -0.01 | [-0.03, 0.00] | -0.02^*^ | [-0.04, -0.001] | Wave 1-3 Couple Subsample | -0.10^*^ | [-0.19, -0.02] |
| Gender × change in porn use over time  (first-difference operator) |  | 0.03^***^ | [0.02, 0.05] | 0.07^***^ | [0.05, 0.08] |  | 0.08^*^ | [0.01, 0.15] |

*Notes*: All variables were standardized; ^***^*p* < .001, ^**^*p* < .01; ^*^*p* < .05.

### **Table S10**

RQ_1-3_ – Alternative predictor: coefficient estimates and 95% CIs of the clustered-adjusted regression models testing the effects of the weekly time spent watching porn as a function of gender (Wave 1) and the fixed-effects panel regression models testing the effects of change in the weekly time spent watching porn as a function of gender (Waves 1-3) on sexual self-competence (RQ_1_), sexual functioning (RQ_2_), and partner-reported sexual satisfaction (RQ_3_).

|  | | **RQ_1_.** Sexual  self-competence | | **RQ_2_.** Sexual functioning | |  | **RQ_3_.** Partner-reported sexual satisfaction | |
| --- | --- | --- | --- | --- | --- | --- | --- | --- |
|  |  | β | 95% CI | β | 95% CI |  | β | 95% CI |
| Gender (-0.5 = men; +0.5 = women) | Wave 1  Sample | -0.08^***^ | [-0.09, -0.08] | -0.13^***^ | [-0.13, -0.12] | Wave 1 Couple Subsample | 0.01 | [-0.02, 0.03] |
| Weekly time spent watching porn |  | 0.01^*^ | [0.00, 0.02] | -0.01^*^ | [-0.02, -0.00] |  | -0.01 | [-0.03, 0.02] |
| Gender × weekly time spent watching porn |  | 0.04^***^ | [0.04, 0.05] | 0.03^***^ | [0.03, 0.04] |  | 0.03^**^ | [0.01, 0.06] |
|  | | β | 95% CI | β | 95% CI |  | β | 95% CI |
| Weekly time spent watching porn | Wave 1-3 Sample | 0 | [-0.01, 0.02] | 0.01 | [-0.00, 0.03] | Wave 1-3 Couple Subsample | -0.06 | [-0.14, 0.03] |
| Gender × weekly time spent watching porn |  | 0.02^***^ | [0.01, 0.03] | 0.03^***^ | [0.02, 0.04] |  | 0 | [-0.08, 0.07] |

*Notes*: All variables were standardized; weekly time spent watching porn was assessed using one item, i.e., “In the past six months, how much time have you spent watching pornography on the Internet?”, where 1 = *0 min* (including people having never intentionally watched porn), 2 = *1-30 min.*, 3 = *30 min. – 1 h* …, and 8 = *3 h+* (*M*_♂_ = 3.29 [*SD* = 1.83], *M*_♀_ = 1.73 [*SD* = 1.09]); ^***^*p* < .001, ^**^*p* < .01; ^*^*p* < .05.

## **Supplementary References**

Lippa, R. A. (2006). Is high sex drive associated with increased sexual attraction to both sexes? It depends on whether you are male or female. *Psychological Science, 17*(1), 46-52.

Perry, S. L. (2018). Pornography use and marital separation: Evidence from two-wave panel data. *Archives of Sexual Behavior, 47*(6), 1869-1880.

Perry, S. L., & Davis, J. T. (2017). Are pornography users more likely to experience a romantic breakup? Evidence from longitudinal data. *Sexuality & Culture, 21*(4), 1157-1176.

Perry, S. L., & Schleifer, C. (2018). Till porn do us part? A longitudinal examination of pornography use and divorce. *The Journal of Sex Research, 55*(3), 284-296.

Snell, W. E. (1998). The multidimensional sexual self-concept questionnaire. In T. D. Fisher, C. M. Davis, W. L. Yarber, & S. L. Davis (Eds.), *Handbook of sexuality-related measures (3rd ed.)* (pp. 537-539).

Štulhofer, A., Buško, V., & Brouillard, P. (2010). Development and bicultural validation of the new sexual satisfaction scale. *Journal of Sex Research, 47*(4), 257-268.
